# Perspectives of Pediatric Cardiology on the Creation of Pediatric Congenital Heart Surgery Subspecialty in Brazil

**DOI:** 10.21470/1678-9741-2024-0200

**Published:** 2025-03-24

**Authors:** Rachel Vilela de Abreu Haickel Nina, Tainá Beiisa Ferreira Rosa, Barbara Neiva Tanaka

**Affiliations:** 1 Hospital Universitário da Universidade Federal do Maranhão (HUUFMA), São Luís, Maranhão, Brazil. E-mail: rachelhaickel@gmail.com; 2 Hospital Universitário da Universidade Federal do Maranhão (HUUFMA), São Luís, Maranhão, Brazil.

We read with great enthusiasm the article entitled “Challenges of Congenital Heart Surgery in Brazil: It is Time to Designate Pediatric Congenital Heart Surgery Subspecialty” which advocates in favor of the creation not only of the specialty Pediatric Heart Surgery but also the creation of training centers for these specialists^[1]^.

The Northeast of Brazil is one of the regions that persists with a lower number of procedures for the treatment of congenital heart diseases (CHD), especially in children under one year of age. In this regard, we see the creation of the subspecialty as an important step to meet part of this need^[2]^. As consequence, the implementation of training centers for pediatric cardiac surgery will certainly create more sites and opportunities for the surgical treatment of these patients.

The Brazilian Constitution and the Child and Adolescent Statute presuppose that health must be offered equally to the entire Brazilian population including children as an important part of this population. Undoubtedly, there is a need to offer child-centered care, and the proposal of the specialty is one of the ways to achieve it^[1]^.

Every day we face lacking timely diagnosis which is not either accepted or understood by family members and is further aggravated by the lack of targeted and specialized treatment for the pediatric population in our country. We deal with enormous regional difficulties and the challenges become even greater when adequately trained professionals are not available.

Brazilian Heart Surgery has exponents in the treatment of CHD, such as Jatene with the correction of transposition of the great arteries, Mendonça with the treatment of coarctation of the aorta, and da Silva with cone operation for the treatment of Ebstein's anomaly. The pioneering spirit of these giant surgeons has influenced and will continue to shape the future of the generations that followed these milestones^[3,4,5]^.

The difficulty and lack of recognition of professionals dedicated to Pediatric Heart Surgery limit not only the implementation of existing treatments but also the development of new techniques. Given the opportunity, we feel happily obliged to raise our voice in favor of this recognition and congratulate the authors for their initiative; we also thank all cardiac surgeons mostly or exclusively dedicated to CHD for their inspirational work.
